# LSPR and Interferometric Sensor Modalities Combined Using a Double-Clad Optical Fiber

**DOI:** 10.3390/s18010187

**Published:** 2018-01-11

**Authors:** Harald Ian Muri, Andon Bano, Dag Roar Hjelme

**Affiliations:** Department of Electronic Systems, Norwegian University of Science and Technology, Gunnerus Gate 1, 7012 Trondheim, Norway; andon2105@hotmail.com (A.B.); dag.hjelme@ntnu.no (D.R.H.)

**Keywords:** reflection-based OF sensor, smart hydrogel, FP interferometer, LSPR, gold nanorods, double-clad optical fiber, multiparameter sensor, single-point sensor

## Abstract

We report on characterization of an optical fiber-based multi-parameter sensor concept combining localized surface plasmon resonance (LSPR) signal and interferometric sensing using a double-clad optical fiber. The sensor consists of a micro-Fabry-Perot in the form of a hemispherical stimuli-responsive hydrogel with immobilized gold nanorods on the facet of a cleaved double-clad optical fiber. The swelling degree of the hydrogel is measured interferometrically using the single-mode inner core, while the LSPR signal is measured using the multi-mode inner cladding. The quality of the interferometric signal is comparable to previous work on hydrogel micro-Fabry-Perot sensors despite having gold nanorods immobilized in the hydrogel. We characterize the effect of hydrogel swelling and variation of bulk solution refractive index on the LSPR peak wavelength. The results show that pH-induced hydrogel swelling causes only weak redshifts of the longitudinal LSPR mode, while increased bulk refractive index using glycerol and sucrose causes large blueshifts. The redshifts are likely due to reduced plasmon coupling of the side-by-side configuration as the interparticle distance increases with increasing swelling. The blueshifts with increasing bulk refractive index are likely due to alteration of the surface electronic structure of the gold nanorods donated by the anionic polymer network and glycerol or sucrose solutions. The recombination of biotin-streptavidin on gold nanorods in hydrogel showed a 7.6 nm redshift of the longitudinal LSPR. The LSPR response of biotin-streptavidin recombination is due to the change in local refractive index (RI), which is possible to discriminate from the LSPR response due to changes in bulk RI. In spite of the large LSPR shifts due to bulk refractive index, we show, using biotin-functionalized gold nanorods binding to streptavidin, that LSPR signal from gold nanorods embedded in the anionic hydrogel can be used for label-free biosensing. These results demonstrate the utility of immobilizing gold nanorods in a hydrogel on a double-clad optical fiber-end facet to obtain multi-parameter sensing.

## 1. Introduction

Sensor miniaturization is an important design objective for biochemical sensors. Size matters, not only for applications requiring insertion into small sample volumes or insertion into tissue or vessels, but also for the functionality of the sensors [1]. Small size means fast diffusion times and therefore fast sensor response. Optical fiber-based sensors represent one popular sensor platform for miniaturization [2,3,4].

Multi-parameter sensing ability is another attractive sensor feature. Many applications require simultaneous measurement of several parameters in a single sample or single position. While this can be achieved using multiple sensor elements, the increased size of the resulting sensor might not be acceptable. Therefore, sensors with multi-parameter sensing ability are receiving increasing attention in the literature [5]. For optical fiber (OF) sensing, one can utilize multiple modes to achieve multi-parameter sensing, for example, in tilted fiber Bragg grating sensors [6,7]. Other techniques include using fluorophores with different fluorescent wavelengths [8,9], or using noble metal nanoparticles with different localized surface plasmon resonance (LSPR) wavelengths [10] in an optrode or on the facet of a cleaved OF. All these sensors achieve multi-parameter sensing utilizing a single sensing modality.

**Figure 1:**
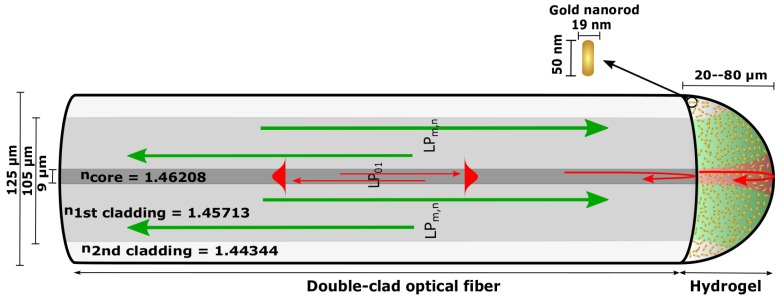
Illustration of the double-clad optical fiber combining interferometric and plasmonic sensor modalities with $n_{\mathrm{core}}=1.46208$, $n_{1\mathrm{st}\;\mathrm{cladding}}=1.45713$, and $n_{2\mathrm{nd}\;\mathrm{cladding}}=1.44344$. Light in the range of 1500–1600 nm ($\lambda_{I}$) is confined as single transverse mode both in the fiber and in the hydrogel volume, with reflection at the OF–hydrogel interface and hydrogel–solution interface illustrated with red color. Multi-mode with light in the range of 450–850 nm ($\lambda_{\mathrm{II}}$) is guided in the first cladding with numerical aperture illustrated with green on fiber-end face. The FP interference is measured with $\lambda_{I}$, while the LSPR signal from gold nanorods (GNR) is measured with $\lambda_{\mathrm{II}}$.

We have recently demonstrated an OF-based multi-parameter sensor concept by combining LSPR and interferometric sensing using a double-clad optical fiber (DCOF) [11,12]. The multi-parameter sensor, as illustrated in Figure 1, consists of a micro-Fabry–Perot (FP) in the form of a hemispherical stimuli-responsive hydrogel [13,14] with immobilized gold nanoparticles (GNPs) on the facet of a cleaved DCOF [15,16].

The swelling degree of the stimuli-sensitive hydrogel is measured interferometrically using the single-mode inner core of the DCOF in the 1500–1600 nm spectral range (noted as $\lambda_{I}$ from now on), while the LSPR signal in the 450–850 nm spectral range (noted as $\lambda_{\mathrm{II}}$ from now on) is measured using the multi-mode (MM) inner cladding of the DCOF. This configuration enables efficient excitation and collection of the LSPR signal using the large numerical aperture (NA) MM waveguide defined by the inner cladding, at the same time as we suppress optical interference in the LSPR spectrum. Similarly, by using the single-mode core of the DCOF we avoid the limitation of modal dispersion in the MM fiber and the fiber-tip FP cavity, and achieve high visibility interference spectra for the interferometric measurement.

In the proof-of-concept experiments reported in [11,12], we demonstrated basic sensor functionality; the visibility of the FP signal was unaffected by the GNP densities up to $1.8\times 10^{11}$ mL^−1^ and particle diameter up to 100 nm. Furthermore, we showed that the LSPR peak wavelength was unaffected by the hydrogel swelling degree. A more detailed study of the LSPR signal from spherical GNPs embedded in anionic hydrogel [15,16] indicated that for the highest particle densities, we had to account for plasmon coupling between proximal GNPs to explain the observed LSPR spectra. Theoretical modelling showed that the plasmon coupling effect could only be explained assuming non-uniform distribution of particles in the hydrogel.

Thus, the effect of embedding spherical GNPs in the hydrogel FP cavity, as well as the feasibility of LSPR-based sensing using GNPs embedded in the hydrogel, have been discussed. However, the effect of GNP shape, hydrogel swelling degree, bulk refractive index (RI), and the feasibility of LSPR-based biosensing in a hydrogel has not been elucidated. In this paper, we characterize the LSPR signal from gold nanorods (GNRs) embedded in an anionic hydrogel. We demonstrate interferometric measurement of hydrogel swelling up to a swelling degree of 6. Hydrogel swelling, induced by pH change, induces a small redshift of the LSPR peak wavelength, rather than the expected blueshift from the reduced averaged RI of the diluted polymer network. The observed redshift can be explained as a result of reduced side-by-side (s-s)-oriented plasmon coupling with hydrogel swelling. Increased bulk RI, using glycerol and sucrose, induces a large blueshift of the LSPR peak wavelength, contrary to the expected redshift from the increased medium RI. This blueshift can only be explained as a result of alteration of the surface electronic structure of the GNRs donated by the anionic polymer network and the glycerol or sucrose solutions [17,18,19,20]. In spite of these unexpected behaviors, we do show, using biotin-functionalized GNRs binding to streptavidin, that the LSPR signal from GNRs embedded in a hydrogel can be used for biosensing.

## 2. Polarizability of GNRs and Hydrogel as Low-Finesse FP Etalon

### 2.1. Fabry-Perot Interferometer

The stimuli-responsive hydrogel shown in Figure 1 represents a low-finesse FP etalon. The FP is interrogated using $\lambda_{I}$ light guided by the single-mode core. Confining the $\lambda_{I}$ light to a single transverse mode, both in the fiber and in the hydrogel volume, ensures effective interference between the field reflected at the fiber–gel interface and the field reflected at the gel–solution interface (as illustrated with red color in Figure 1).

The optical length $l_{0}$ and the length change $\Delta l_{0}$ can be estimated from the wavelength-dependent FP reflection. Both the reflection at the gel–solution interface, $r_{2}$, and at the fiber–gel interface, $r_{1}$, are small, such that the reflected intensity of the hydrogel FP can be approximated as

(1) $$I_{\mathrm{FP}}\left(\lambda \right)=I_{0}\left[{r_{1}}^{2}+{\left(\gamma r_{2}\right)}^{2}+2\gamma r_{1}r_{2}\cos\left(\frac{4\pi l_{0}}{\lambda }+\varphi_{0}\right)\right]$$

where $k=\frac{2\pi }{\lambda }$, γ is a loss factor (due to absorption, scattering and mode mismatch), and $\varphi_{0}$ is the initial arbitrary phase. The free spectral range (FSR) is related to the optical length as

(2) $$\mathrm{FSR}=\frac{{\lambda_{0}}^{2}}{2l_{0}}$$

where $\lambda_{0}$ is the wavelength of observation. The length change is related to the phase change of the interferometric spectrum as

(3) $$\Delta \phi =\frac{4\pi \Delta l_{0}}{\lambda_{0}}$$

The change in $l_{0}$ may originate from both a change in RI of the gel and from a change in the physical length *l* of hydrogel cavity,

(4) $$\Delta l_{0}=\Delta ln_{\mathrm{gel}}+l\Delta n_{\mathrm{gel}}$$

where $n_{\mathrm{gel}}$ is the RI of the gel, originating from both the solvent and the polymer concentration.

Over the short propagation distances used here (a few meters), the mode-coupling between the single-mode (SM) core and the MM cladding will be negligible. The crosstalk between the MM channel and the SM channel in the fiber coupler is not specified. However, since we are using spectrally resolved detection in both channels, we would not be affected by any coupler crosstalk.

### 2.2. LSPR of GNRs in Hydrogel

The LSPR of the GNRs in the hydrogel is probed using $\lambda_{\mathrm{II}}$ light guided in the MM-first cladding shown in Figure 1. By using the high NA of the large-diameter core for the $\lambda_{\mathrm{II}}$ light, we ensure effective excitation of a large fraction of the GNRs immobilized in the hydrogel volume and effective collection of the reflection from the LSPR of the GNRs (as illustrated with green color in Figure 1). The reflection from the GNRs in hydrogel on the OF end face is a result of the extinction that is the sum of scattering and absorption. With a sufficiently low GNR density, and absence of dipole–dipole interactions, the optical properties of GNRs in hydrogel can be described by Gans theory, a generalization of Mie theory for spheroidal particles [21,22]. In the quasi-static approximation, the polarizability of the longitudinal plasmon mode of GNRs is

(5) $$\alpha =\frac{(1+\kappa )V}{4\pi }\left(\frac{\varepsilon \left(\lambda \right)-\varepsilon_{m}}{\varepsilon \left(\lambda \right)+\kappa \varepsilon_{m}}\right)$$

(6) $$\kappa =\frac{1-L}{L}$$

(7) $$L=\frac{1-e^{2}}{e^{2}}\left[\frac{1}{2e}\ln\left(\frac{1+e}{1-e}\right)-1\right]$$

(8) $$e=\sqrt{1-{\left(\frac{1}{\mathrm{AR}}\right)}^{2}}$$

where AR is the aspect ratio of the GNRs, $\varepsilon \left(\lambda \right)=\varepsilon_{1}+i\varepsilon_{2}$ is the complex metal dielectric function, and *V* is the volume of the GNRs.

The polarizability is maximized when the real part of the metal dielectric function, $\varepsilon_{1}\left(\lambda \right)$, and the dielectric constant of the surrounding medium, $\varepsilon_{m}$, satisfy the relation

(9) $$\varepsilon_{1}\left(\lambda \right)=-\kappa \varepsilon_{m}$$

By using the Drude model, the LSPR peak position as a function of local RI, $n_{m}=\sqrt{\varepsilon_{m}}$, can be described as

(10) $$\lambda_{\max}=\lambda_{p}\sqrt{\kappa {n_{m}}^{2}+1}$$

where $\lambda_{p}$ is the plasma oscillation wavelength of the bulk metal [23]. For the transverse resonance, we have to replace κ with $\kappa_{T}=\frac{1+L}{1-L}$.

For GNRs in close proximity to each other, the LSPR of the longitudinal plasmon mode will change with decreasing center-to-center interparticle distance *d* [24]. For two identical GNRs, the resonance permittivities of the dipole–dipole coupling for the longitudinal plasmon mode can be described by the point dipole model [25]. For the side-by-side (s-s) or the end-to-end (e-e) cluster configuration, the plasmon resonance condition becomes

(11) $$\varepsilon_{1}{\left(\lambda \right)}_{\left(\pm \right)}=-F_{\left(\pm \right)}\varepsilon_{m}$$

(12) $$F_{\left(\pm \right)}=-\left(\frac{g^{2}+\kappa }{g^{2}-1}\pm \frac{g(\kappa +1)}{g^{2}-1}\right)$$

(13) $$g=\frac{N(1+\kappa )V}{4\pi d^{3}}$$

where $F_{\left(+\right)}$ with N=2 represents the e-e configuration and $F_{\left(-\right)}$ with N=1 represents the s-s configuration (dipole moment maximized with $\frac{\alpha^{2}}{d^{6}}=1$ for the s-s and with $\frac{4\alpha^{2}}{d^{6}}=1$ for the e-e configuration). The LSPR peak position as a function of $n_{m}$ and *d* can be further expressed by using the Drude model,

(14) $$\lambda_{\max(\pm )}=\lambda_{p}\sqrt{F_{\left(\pm \right)}{n_{m}}^{2}+1}$$

**Figure 2:**
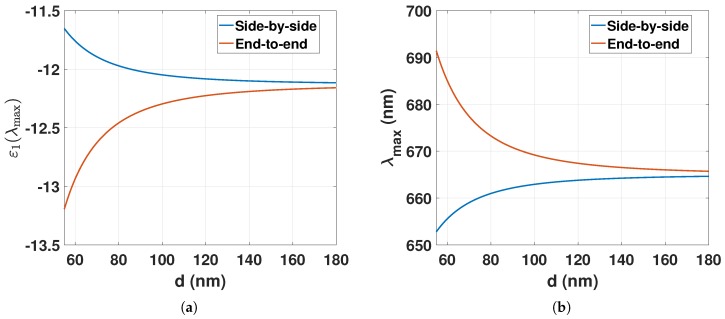
(**a**) Resonance permittivities (Equation (11)) of two identical GNRs in the s-s or e-e configuration for decreasing *d*; (**b**) $\lambda_{\max}$ (Equation (14)) of two identical GNRs in the s-s or e-e configuration for decreasing *d*. $\varepsilon_{m}=n_{m}^{2}=1.33^{2}$, $\lambda_{p}=183$ nm, GNR width = 19 nm, GNR length = 50 nm, and AR=50/19.

The plasmon resonance condition and $\lambda_{\max}$ can be computed from Equation (11) and (14), as shown in Figure 2 for decreasing *d*. In the e-e configuration, a decrease in interparticle distance, *d*, will redshift the LSPR peak position, whereas in the s-s configuration a decrease in *d* will result in a blueshift.

Both absorption and scattering will contribute to the reflected LSPR signal. If we ignore interference effects (see discussion below) and assume for simplicity that scattering and absorption are weak, we can approximate the change in reflected power due to the GNRs as

(15) $$\Delta P_{R}={\left(1-R_{1}\right)}^{2}\left[\frac{1}{2}{\left(\frac{\mathrm{NA}}{4}\right)}^{2}\sigma_{\mathrm{sc}}-R_{2}\sigma_{\mathrm{ex}}\right]\rho 2lP_{\mathrm{in}}$$

where $R_{1}$ is the reflectance of the fiber–hydrogel interface, $R_{2}$ is the reflectance of the hydrogel–solution interface, ρ is the GNP density, and $P_{\mathrm{in}}$ is the input power. Depending on the relative strength of the scattering and extinction terms, we will either observe an LSPR reflection dip (net absorption) or a LSPR reflection peak (net scattering). For the particle sizes and densities, and hydrogel compositions used in this work, we observe a net absorption.

The FP interferences will not be observable using light propagating in the MM core due to modal dispersion in the OF cable and in the FP cavity. Assuming the propagation in the FP cavity can be described using Hermite-Gaussian beams with indexes (l,m), the higher-order modes ((l,m)>(0,0)) propagate with an excess phase $\left(l+m\right)\tan^{-1}\left(\frac{z}{z_{0}}\right)$, where $z_{0}$ is the Rayleigh range [26]. The DCOF carries several thousand modes (V-parameter is approximately 100) resulting in the same number of Hermite-Gaussian modes in the FP cavity. Therefore, the visibility of the FP interference will effectively be reduced to zero and will not be observable. Adding modal dispersion in the OF will further reduce the visibility.

## 3. Materials and Methods

Two sets of experiments were performed with two different GNR–hydrogel fiber-optic (FO) sensors; (1) the FSR and LSPR peak positions were measured as a function of pH and RI; (2) the LSPR peak positions were determined for nonfunctionalized GNRs in hydrogel, biotin-functionalized GNRs in hydrogel, and for the biotin–streptavidin recombination on the surface of the GNRs immobilized in the hydrogel.

### 3.1. Fabricating the GNR–Hydrogel

The FO sensors were fabricated as described from previous work [15,16]. Citrate-stabilized 670 nm-resonant GNRs (50 nm in length, 19 nm in diameter, $1.14\times 10^{13}$ particles/mL, 2 mM citrate buffer, nanoCompix) were used to make pregel solutions of 10 wt % acrylamide (AAM)–acrylic acid (AAC) (molar ratio 1/2 AAM/AAC) and 2 mol % *N*, *N*-methylenebisacrylamide (BIS).

### 3.2. Setup of the FO Sensor Instrument

**Figure 3:**
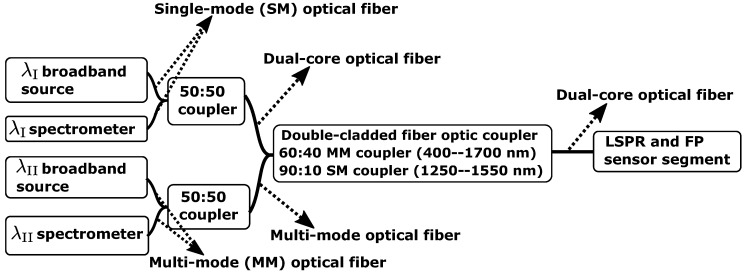
Setup of the fiber-optic instrument based on reflection measurements.

The FO-sensor setup illustrated in Figure 3 consist of the following components; $\lambda_{\mathrm{II}}$ broadband source (MBB1F1, 470–850 nm, Thorlabs, Newton, NJ, USA), $\lambda_{I}$ broadband source (S5FC1005S, 1550 nm, 50 nm bandwidth, Thorlabs), 50:50 coupler MM (50/50, FCMH2-FC, 400–1600 nm, Thorlabs), 50:50 coupler SM (50/50, 84075633, 1550 nm, Bredengen, Oslo, Norway), double-clad optical fiber (DCOF) coupler (DC1300LEB, MM 400–1700 nm, SM 1250–1550 nm, Thorlabs), $\lambda_{\mathrm{II}}$ spectrometer (QE65Pro, Ocean Optics, Dunedin, FL, USA), $\lambda_{I}$ spectrometer (NIRQuest-512-1.7, Ocean Optics), loose fiber-end terminated with index-matching gel (G608N3, Thorlabs), LSPR and FP sensor segment with Ø125 µm DCOF (DCF13, Thorlabs).

Data acquisition was performed with the Spectrasuite program software (Ocean Optics) and the OFs were spliced using a Fitel Fusion Splicer (Furukawa Electric, Yokohama City, Japan).

### 3.3. Preparation of Solutions for Hydrogel Swelling and LSPR Shifts

Hydrochloric acid (HCl) or sodium hydroxide (NaOH) (1.0 M, Sigma Aldrich, St. Louis, MO, USA) were added to Milli-Q water to prepare pH solutions to stimulate a change in volume of the hydrogel. pH was controlled with a pH meter (inoLab pH/ION 7320, WTW, New York, NY, USA), pH electrode (pHenomenal MIC 220, Van Water & Rogers (VWR) Collection, Radnor, PA, USA), and temperature sensor (pHenomenal TEMP21, VWR Collection). Glycerol (>99%, VWR) or Sucrose (>99.5%, VWR) were added to Milli-Q water to prepare RI solutions to induce shift in the LSPR peak position. The bulk RI values for different wt % (between 0 and 40 wt %) of glycerol and sucrose in Milli-Q water were obtained from the Handbook of Chemistry and Physics at λ = 589 nm [27].

### 3.4. Functionalizing GNRs in Hydrogel with Biotin for Biotin–Streptavidin Recombination

#### 3.4.1. Functionalizing GNRs in Hydrogel with Biotin

Biotin with polyethylene glycol (PEG) chains terminated with a thiol group (Biotin-PEG (5k)-SH, powder, PG2-BNTH-5k, NANOCS Inc., New York, NY, USA) were reconstituted in Milli-Q solution to 0.01 M. Further, tris(2-carboxyethyl)phosphine hydrochloride (TCEP, powder, Sigma Aldrich) with 10 molar equivalents to biotin-PEG(5k)-SH were added to the 0.01 M biotin solution and stirred for 10 min to ensure that disulfide bonds were reduced to thiol groups. Next, the GNR–hydrogel on OF was immersed in the biotin solution for 10 min to let the thiol group bind to the GNRs. Last, the GNR–hydrogel on OF was transferred to Milli-Q water solution with pH 4.5 to remove any residues of biotin in the hydrogel.

#### 3.4.2. Biotin–Streptavidin Recombination on GNRs in Hydrogel

Streptavidin (Streptomyces avidinii, powder, S4762, Sigma Aldrich) was reconstituted in phosphate-buffered solution (PBS, Sigma Aldrich) to a concentration of 1 mg/mL and readjusted to a pH of 4.5. The fiber–gel with biotin-functionalized GNRs was then immersed in the streptavidin solution for 10 min and transfered to Milli-Q water solution with pH 4.5.

### 3.5. Reflection Measurements of GNRs Embedded in Hydrogel in $\lambda_{\mathrm{II}}$ and $\lambda_{I}$

The reflectance spectra were estimated from the measured raw spectra, $S_{\lambda }$, normalized to a measured reference spectra, $R_{\lambda }$. Before normalization, we subtracted the measured dark spectrum, $D_{\lambda }$ (recorded with the light source turned off), from both the raw spectra and a reference spectrum. The normalized reflectance spectra were then computed as

(16) $$I_{R}=\left(\frac{S_{\lambda }-D_{\lambda }}{R_{\lambda }-D_{\lambda }}\right)$$

The hydrogel swelling, or deswelling, was induced by immersing the hydrogel–fiber in solutions with pH between 4.5 and 3.0. The change in LSPR was induced by immersing the hydrogel–fiber in glycerol or sucrose solutions with bulk RI between 1.330 and 1.385 at pH 4.5 and 3.0. For the biotin–streptavidin measurements, all spectra were recorded in Milli-Q water with pH at 4.5. For each solution, the hydrogel was left for 1 minute to reach equilibrium before sampling the spectrum. For convenience, we used different reference spectra for the different experiments:

- For the FP experiments, we used the reflection spectrum of the bare DCOF in Milli-Q water solution.
- For the LSPR experiments, we used the reflection spectra from the hydrogel without GNRs for each pH, glycerol and sucrose solution, to compensate for the artefacts in the LSPR spectra caused by the reflections at the fiber–gel interface.

### 3.6. Estimating the FSR and the LSPR Peak Position

We determined the FSR from the autocorrelation function of the reflectance spectra to relax the dependence on signal normalization. The autocorrelation function is symmetric and measures the correlation between $I_{R\left(i\right)}$ and $I_{R(i+k)}$ for lag time *k* = 0, 1, 2 … (*N*−1), where *N* is the length of the vector received from the spectrometer. The autocorrelation coefficients for lag time *k* are described as

(17) $$r_{k}=\frac{\sigma^{2}}{N-1}\sum_{i=1}^{N-k}\left(I_{R\left(i\right)}-\bar{I_{R}}\right)\left(I_{R(i+k)}-\bar{I_{R}}\right)$$

where $\bar{I_{R}}$ is the mean of $I_{R}$, and $\sigma^{2}$ is the sample variance of the lag time-series [28]. To find the FSR, the centered and scaled smoothing spline function was applied on the first peak of the autocorrelation function with a smoothing parameter at 0.99. With smoothing parameter p=0, the smoothing spline function produces a least-squares line fit to the data, whereas with p=1, the smoothing spline function produces a cubic spline interpolant. By choosing a fixed smoothing parameter, the balance between residual error and local variation is also fixed [29].

For $\lambda_{\mathrm{II}}$ reflectance measurements of GNPs, the LSPR spectrum was fitted with a centered and scaled smoothing spline function with smoothing parameter at 0.995.

## 4. Results

The quality of the interferometric and LSPR signals were first assessed and compared to previous work. Secondly, the FSR and the LSPR were measured as a function of hydrogel swelling degree to assess the influence of GNRs on the interferometric signal and to assess the influence of hydrogel deswelling on LSPR, respectively. Thirdly, the LSPR response and optical length were measured as a function of RI solutions. In addition, the LSPR peak positions were determined for constant and dynamic reference spectra for pH and RI measurements to determine the influence of the change in reflections at the gel–fiber interface on the LSPR signal. Last, the LSPR peak position was determined for the biotin–streptavidin recombination on GNRs in hydrogel.

### 4.1. Acquisition of LSPR and Interferometric Signals

**Figure 4:**
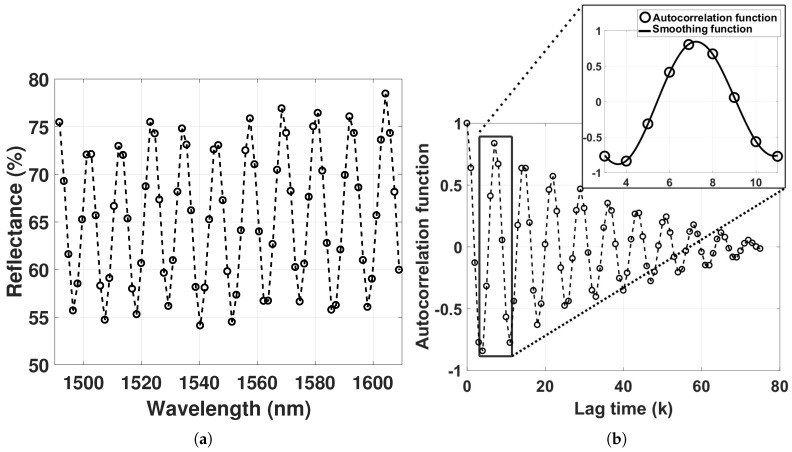
(**a**) Reflectance in the $\lambda_{I}$ range from the hydrogel with GNRs in pH of 4.5; (**b**) autocorrelation function of the interferometric spectrum.

The $\lambda_{I}$ reflectance spectrum from a GNR–hydrogel in pH 4.5, together with the resulting autocorrelation function of the reflectance spectrum used to find the FSR, are shown in Figure 4.

The smallest FSR in our experiments, at pH 4.5, corresponds to optical lengths of the hydrogel of around 100 µm, with visibility of approximately 0.15. Aggregation of GNRs in the hydrogel would significantly reduce the visibility of the interferogram. Thus, the degree of GNR aggregation can be assessed by monitoring the quality of the interferogram. The visibility in Figure 4a is, however, comparable to previously fabricated extrinsic Fabry–Perot interferometers (EFPI) despite using a GNR particle density of $1.14\times 10^{13}$ mL^−1^ [13] compared to $1.9\times 10^{11}$ mL^−1^ used in our earlier work [11,12].

**Figure 5:**
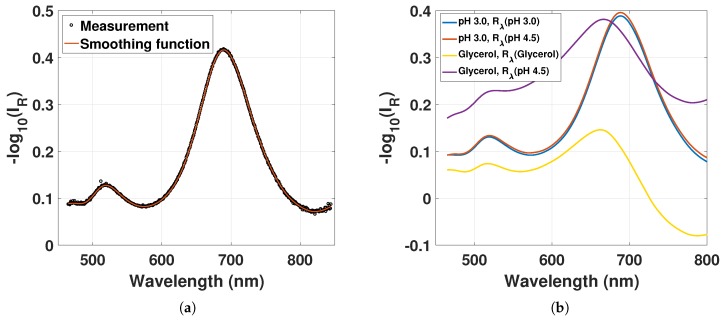
(**a**) Reflectance from the GNR-hydrogel with smoothing function, in pH 4.5 with $R_{\lambda }\left(\mathrm{pH}\;4.5\right)$; (**b**) reflectance from the GNR-hydrogel in (1) pH 3.0 with $R_{\lambda }\left(\mathrm{pH}\;3.0\right)$ and $R_{\lambda }\left(\mathrm{pH}\;4.5\right)$ and (2) 40 wt % glycerol at pH 4.5 with $R_{\lambda }$ (40 wt % glycerol, pH 4.5) and $R_{\lambda }\left(\mathrm{pH}\;4.5\right)$.

The measured LSPR reflectance spectrum from the GNR–hydrogel in pH 4.5 shows a transverse plasmon mode at 519 nm and a longitudinal plasmon mode at 689 nm (Figure 5a). Due to the MM propagation and multi-transverse-mode hydrogel FP cavity, no interference fringes are observed. The longitudinal LSPR in Milli-Q water at pH 4.5 is redshifted by 19 nm compared to the LSPR in the citrate-buffered solution, due to the increased RI of the polymer network compared to the citrate-buffered solution.

The LSPR reflectance spectrum in Figure 5a rides on top of an 82% background dominated by the wavelength-independent Fresnel reflection at the fiber-hydrogel interface. In addition, there will be a small wavelength-dependent reflection from the hydrogel, both scattering from hydrogel inhomogeneities and from the hydrogel-solution interface. This signal contribution will also be modulated by any spectrally dependent propagation absorption in the hydrogel. As discussed in Section 3.5, to account for this, the LSPR spectrum is found by normalizing the measured reflected intensity from the GNR-hydrogel to the reflected intensity from another hydrogel without GNRs exposed to the same solution. However, due to sensor-to-sensor variation in hydrogel preparation, this normalization procedure is prone to errors. To explore this, we have quantified the effect of using a fixed reference spectrum for all LSPR reflectance spectra. As we can see from Figure 5b, for the sensor in Milli-Q water with varying pH, the effect of keeping the reference spectrum constant is insignificant.

This is due to the insignificant variation in RI with varying pH. On the other hand, in the experiments using solutions with glycerol (or sucrose), the effect of keeping the reference spectrum constant is large, as we see from the two glycerol traces in Figure 5b. The largest effect is due to the reduced Fresnel reflection at the fiber–hydrogel interface with increasing solution RI (increasing glycerol). However, the resulting shift in the estimated LSPR is moderate, since the reflection spectra from the hydrogel without GNRs are practically constant in the wavelength range of the longitudinal LSPR wavelength. These variations of the LSPR signal for constant or variable reference spectra are further discussed in Section 4.3. We define a resonant wavelength “error” as the resulting wavelength shift due to variation in the reference spectra used.

The figure of merit is expected to be larger for the longitudinal plasmon mode than the transverse plasmon mode. We have therefore used the longitudinal plasmon mode in both pH and RI measurements.

### 4.2. FSR Response for pH and RI

**Figure 6:**
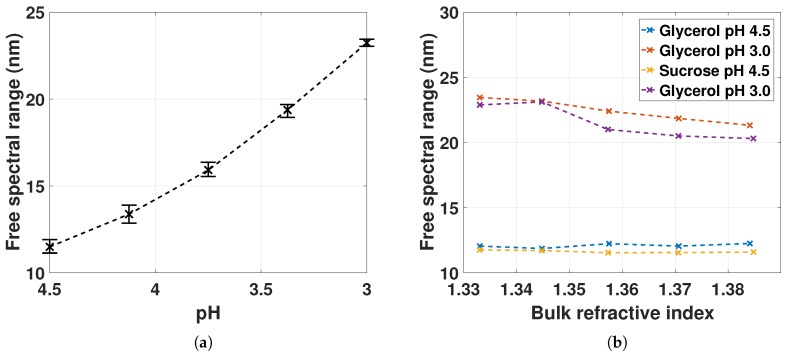
(**a**) FSR measured for the hydrogel deswellling from pH 4.5 to 3.0 for two sampled series with mean, minimum and maximum values from 4 sampled FSRs; (**b**) FSR measured for increasing bulk RI with pH 4.5 and 3.0 for one sampled series.

Figure 6 shows the measured FSR as a function of pH and bulk RI. We note that the GNRs do not disrupt the FSR measurements. The sensitivity of the interferometric sensor should therefore be similar to previous work on EFPI and sufficient for many applications [13]. The sensitivity can, however, be improved by detecting the change in phase, in addition to measuring the FSR [13,14].

The FSR as a function of increasing bulk RI prepared with glycerol and sucrose shows to have small variations at pH 4.5. At pH 3.0 there is, however, a slight decrease in FSR for increasing bulk RI. With initial FSR of 12 nm and 22 nm, the resulting change in optical lengths are 3.76 µm and 1.88 µm (from Equation (4)), respectively, for increasing RI from 1.33 to 1.38. A change in optical length of 3 µm due to increased RI will result in a 1.5 nm change in FSR ($\frac{\Delta \mathrm{FSR}}{\mathrm{FSR}}\propto \frac{\Delta l_{0}}{l_{0}}$ and $l_{0}=50$ µm). From Figure 6b, the change in FSR is, however, larger than this at pH 3.0. Thus, the decrease in FSR at pH 3.0 is most likely a result of the hydrogel swelling due to increased wt % of glycerol or sucrose.

### 4.3. LSPR Response for pH and RI

**Figure 7:**
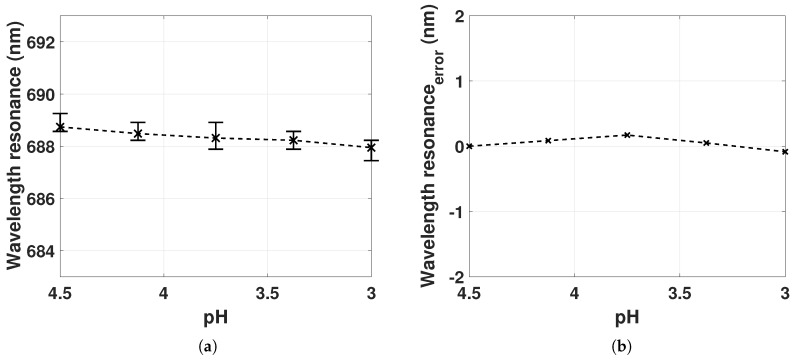
(**a**) LSPR peak position measured for the hydrogel deswelling from pH 4.5 to 3.0 for two sampled series with mean, minimum and maximum values from 4 sampled LSPR peak positions; (**b**) the error of the LSPR peak position by holding the reference spectrum constant at $R_{\lambda }\left(\mathrm{pH}\;4.5\right)$ for pH 4.5 to 3.0. RI = 1.33 (Milli-Q water).

Figure 7a shows the LSPR peak position as a function of pH from 4.5 to 3.0. The mean LSPR peak position for hydrogel deswelling shows a blueshift of 0.8 nm. Variations in the LSPR peak position can occur as a result of change in polymer density of the hydrogel or change in the interparticle distances between the GNRs, inducing electromagnetic interactions between longitudinal plasmon modes. As described in Section 2.2, decreasing interparticle distances for GNRs in an e-e configuration leads to an LSPR redshift, whereas for GNRs in an s-s orientation, decreasing interparticle distance leads to an LSPR blueshift. The observed blueshift of the mean LSPR peak position with hydrogel deswelling suggests increasing dipole–dipole interactions for a large fraction of the GNRs in the s-s configuration. The large fraction of GNRs in the s-s configuration might be due to many factors, for example, the polymerization process or the negatively charged polymer network. Investigations concerning the orientation of GNRs in hydrogel are works in progress. The variations of the LSPR peak positions for each pH value suggest that there is a weak dependence on the polymer density. This weak dependence is likely due to the low polymer density of the gel, as well as the small dimension of the RI “probes” of GNRs with size of ∼50 nm.

The estimated LSPR wavelength error due to variations in the reference spectra (Equation (16)) for decreasing pH is shown in Figure 7b. The estimate is computed from the measured mean LSPR peak position using constant $R_{\lambda }\left(\mathrm{pH}\;4.5\right)$ subtracted from the mean LSPR peak position from Figure 7a. The LSPR reflection spectrum concerning the background intensity was also discussed in Figure 5. The small wavelength resonance error for decreasing pH demonstrates that the change in background intensity (Fresnel reflection coefficients) from the fiber–gel interface introduces negligible change in the LSPR peak position when keeping the reference spectrum constant at $R_{\lambda }\left(\mathrm{pH}\;4.5\right)$. Thus, despite the large variations in the optical length of a hydrogel cavity, it is feasible to use one reference spectrum from the hydrogel without GNRs in the $\lambda_{\mathrm{II}}$ range.

**Figure 8:**
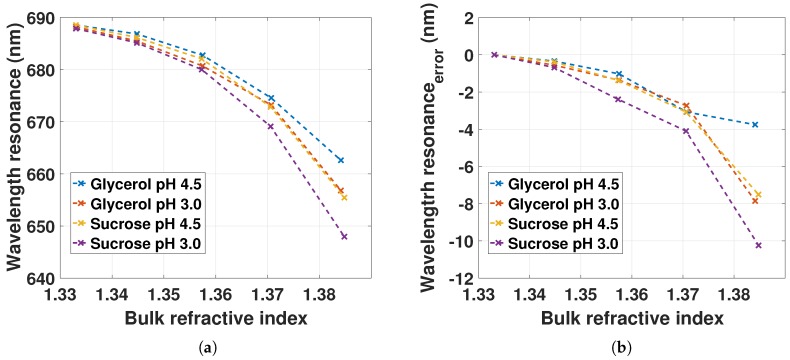
(**a**) LSPR peak position as a function of bulk RI with pH 4.5 and 3.0 for one sampled series; (**b**) the error of the LSPR peak position by holding the reference spectrum constant at $R_{\lambda }\left(\mathrm{pH}\;4.5\right)$ for increasing bulk RI with pH 4.5 and 3.0.

Figure 8a shows the LSPR peak position measured for increasing bulk RI at pH 4.5 and 3.0. The LSPR peak position is blueshifting with a nonlinear trend for increasing bulk RI. The blueshifts at pH 3.0 are larger than at pH 4.5 for both glycerol and sucrose solutions. The LSPR response is also different for glycerol and sucrose solutions. The total change in LSPR peak position for the change in RI is ∼677 nm/RIU. This is in contrast to the expected linear redshift for a LSPR RI probe (from Equation (10) the redshift should be approximately 500 nm/RIU). The RI on the outside and the inside of the GNR–hydrogel is expected to be similar, since glycerol and sucrose are water soluble, and therefore solvents for the hydrogel. The observed blueshift must therefore be due to the nature of the local surrounding medium [17,18,19,20]. In previous work, the optical properties of cetylpyridinium chloride-stabilized gold nanoparticles when exposed to various solvents were studied [19]. The LSPR peak position was shown to be greatly influenced by the properties of the solvent and the removal or donation of electron density onto gold particles. In another study, the LSPR peak position of chitosan-stabilized gold nanoparticles exhibited a blueshift for the specific detection of increasing concentration of mercury in liquid solutions [20]. The blueshift observed in Figure 8a for increasing bulk RI could then be a result of the alteration of the surface electronic structure of the GNRs donated by the charged AAM–AAC polymer network and the glycerol or sucrose solutions. At pH 4.5, the hydrogel has a large fraction of negatively charged AAC, as opposed to the small fraction at pH 3.0. The increase in LSPR blueshift from pH 4.5 to pH 3.0 could be due to the decrease in the negative charge of the gel, changing the donation of electron density onto the GNRs. In addition, the sucrose solutions decrease the LSPR peak position compared to the glycerol solutions. This is also an indication of a change in the solvent properties of the water in gel that is different for glycerol and sucrose.

The LSPR wavelength error for increasing bulk RI is presented in Figure 8b. It is computed from the measured LSPR peak position with constant $R_{\lambda }\left(\mathrm{pH}\;4.5\right)$ subtracted the LSPR peak position from Figure 8a. The uncertainty from the change in reflection at the fiber–gel interface is increasing for increasing bulk RI. This is likely caused by the change in background intensity from the fiber–gel interface as a result of the change in Fresnel reflection coefficients for increasing glycerol or sucrose wt %. The uncertainty values are similar for increasing bulk RI up to 1.37 for all solutions, except for sucrose at pH 3.0. The Fresnel reflection coefficients at the fiber–gel interface are therefore similar for all solutions up to RI of 1.37, except for sucrose at pH 3.0.

The maximum estimated LSPR wavelength error (up to 10 nm) is still smaller than the total LSPR blueshift observed in Figure 8a. The blueshift is therefore not due to the uncertainties in the reference spectra used in our measurements. The study of the influence of the hydrogel and solvents on the LSPR of gold nanospheres or rods is a work in progress.

Note that for label-free biosensing with LSPR, it is only the surface of the GNRs that senses changes in RI. The Fresnel reflection coefficients at the fiber–gel interface are therefore not expected to change upon receptor–analyte recombination. The results obtained in Figure 6, Figure 7 and Figure 8 therefore prove the utility of immobilizing GNRs in hydrogel to measure both interferometric and LSPR signals with acceptable levels of crosstalk [14,30].

### 4.4. Biotin–Streptavidin Recombination on GNRs in Hydrogel

**Figure 9:**
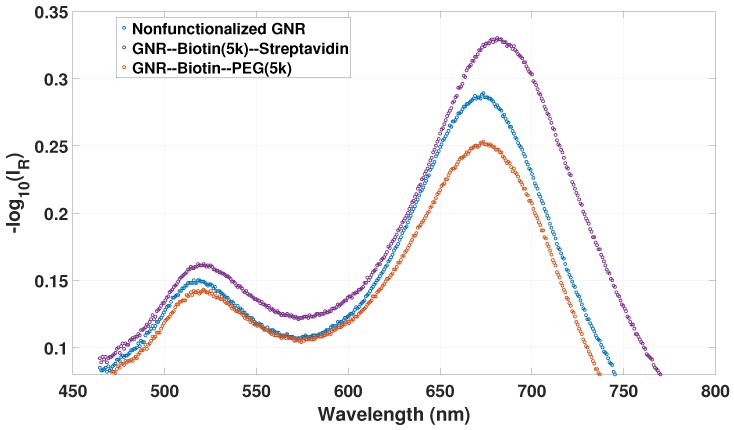
$\lambda_{\mathrm{II}}$ reflectance spectra for bare GNRs, biotin-functionalized GNR and biotin–streptavidin recombination on GNRs in hydrogel in Milli-Q water with pH at 4.5.

Figure 9 shows the $\lambda_{\mathrm{II}}$ reflectance for nonfunctionalized GNRs, biotin-functionalized GNRs and biotin–streptavidin recombination on GNRs in hydrogel in Milli-Q water with pH at 4.5. The transverse and longitudinal LSPR peak positions of the nonfunctionalized GNRs are at 518.75 and 672.35 nm, respectively. The LSPR peak position deviates from the peak positions for the FO sensor used for the pH and RI measurements. The difference in LSPR peak positions obtained for different fabricated OF sensors may be due to the different RI “probing” of the polymer network due to the polymerization process resulting in polymer chains in close proximity to the plasmonic waves on the GNRs.

By functionalizing the GNRs with biotin, the transverse LSPR redshifts by 1.52 nm, whereas the longitudinal LSPR redshifts by 1.45 nm. The changes in transverse and longitudnial LSPRs indicate that the biotin–PEG thiol group is distributed on both the sides and ends of the GNRs. Recombination of biotin–streptavidin on the GNRs changes the transverse and the longitudinal LSPRs by another 0.76 and 7.62 nm, respectively, which also indicates that streptavidin is distributed on both the sides and ends. The interferometric signal was unstable during the biotin functionalization due to the low pH, around 2, of the biotin solution, but recovered approximately to the initial signal and initial FSR value after the functionalization in solution of pH 4.5. During streptavidin recognition, the interferometric signal and FSR were approximately constant.

The 7.6 nm redshift for the streptavidin recognition is similar to previous studies on the LSPR response of GNR towards analytes [31]. The ratio between the total shift of the longitudinal and the transverse plasmon mode is 3.97 (9.05 nm/2.28 nm). From Equation (10) we estimate the expected longitudinal-to-transverse LSPR shift ratio to be 4.1 ($\frac{\partial \lambda_{L}}{\partial \lambda_{T}}=\frac{\kappa_{L}\lambda_{T}}{\kappa_{T}\lambda_{L}}$, AR = 2.63, $\kappa_{L}=\frac{1-L}{L}$, $\kappa_{T}=\frac{1+L}{1-L}$, $\kappa_{L}=6.85$, $\kappa_{T}=1.29$), which is consistent with the observed shift ratio.

While the LSPR in Figure 8a is dependent on the RI and the solvent attributes of increasing wt % of glycerol or sucrose within the gel, the LSPR in Figure 9 is only dependent on the change in RI on the surface of the GNRs in the hydrogel due to the biotin–streptavidin recombination. As the discrimination is made between the change in local RI and bulk RI from the measurements obtained in Figure 8a and Figure 9, this indicates that the blueshift for increasing glycerol or sucrose is a result of the solvent properties of water–glycerol or water–sucrose and the gel, changing the donation of electron density onto the GNRs as discussed in Section 4.3.

### 4.5. Summary of Results

We have created a summary of the results, shown in Table 1 and Table 2, to present an overview of the main findings.

## 5. Conclusions

We have reported on the characterization of an optical fiber-based multi-parameter sensor concept based on combining the LSPR signal and interferometric sensing using a double-clad optical fiber. The sensor consists of a micro-Fabry–Perot in the form of a hemispherical stimuli-responsive hydrogel with immobilized gold nanorods on the facet of a cleaved fiber. The swelling degree of the hydrogel is measured interferometrically using the single-mode inner core, while the LSPR signal is measured using the multi-mode inner cladding.

We have explored the effect of hydrogel swelling and variation of bulk solution RI on the LSPR peak wavelength, demonstrating that pH-induced hydrogel swelling causes only weak redshifts of the longitudinal LSPR mode of the nanorods, while increased bulk RI using glycerol and sucrose causes large blueshifts. The redshifts with hydrogel swelling are likely due to the reduced plasmon coupling in the side-by-side configuration, as the interparticle distance increases with increasing swelling. The total redshift is less than 1 nm for an optical cavity length change from 55 µm to 100 µm, corresponding to a volume-swelling degree of 6. Thus, the variations of the LSPR peak position will be negligible for optical length changes within a smaller range in the volumetric measurements of stimuli-responsive hydrogel. The blueshifts with increasing bulk RI are likely due to alteration of the surface electronic structure of the GNRs donated by the charged AAM–AAC polymer network and glycerol or sucrose solutions. Therefore, care must be taken when using this sensor concept for bulk RI sensing. Both the hydrogel properties and the molecular species causing the RI change must be accounted for.

To explore the feasibility of using the sensor concept for biosensing, that is, measuring the local RI change due to binding to receptors at the GNR surfaces, we used the biotin–streptavidin system. The recombination of biotin–streptavidin on GNRs in hydrogel in Milli-Q water at pH 4.5 showed a 7.6 nm redshift of the longitudinal LSPR. The LSPR response of biotin–streptavidin recombination is due to the change in local RI, which is possible to discriminate from the LSPR response due to changes in bulk RI.

The quality of the interferometric signal is comparable to previous work on EFPI hydrogel-based FO systems, despite having GNRs immobilized in the gel. The FSR increased monotonically for hydrogel deswelling controlled with pH solution, demonstrating the feasibility of utilizing stimuli-responsive hydrogel containing GNRs for label-free sensing. Detecting the phase of the interferometric signal in addition to the FSR would further improve the sensitivity [13].

The FSR and LSPR measurements of the hydrogel swelling degree and biotin–streptavidin recombination prove the utility of immobilizing GNRs in hydrogels to measure both interferometric and LSPR signals with acceptable levels of crosstalk for use in, for example, medical applications [14,30]. Further work will consist of realizing the LSPR and interferometric FO system as a biosensor towards medical applications where specific markers will be detected [14,30,32,33]. The influence of hydrogel and solvent on gold nanospheres and nanorods will also be studied further, in terms of characterizing their configuration and LSPR response.

## Figures and Tables

**Table 1:** Summary of results for interferometric sensor in the $\lambda_{I}$ range.

Stimuli	Free Spectral Range Shift	Mechanism
Hydrogel deswelling	Large increase	Decreased physical length
Bulk refractive-index increase	Small decrease	Hydrogel swelling due to solvent

**Table 2:** Summary of results for LSPR sensor in the $\lambda_{\mathrm{II}}$ range.

Stimuli	LSPR Shift	Mechanism
Hydrogel deswelling	Small blueshift	Increased plasmon coupling
Bulk refractive-index increase	Large blueshift	Change in local surrounding media
Analyte binding to receptors	Redshift	Local refractive index increase
